# The role of transient receptor potential vanilloid type-2 ion channels in innate and adaptive immune responses

**DOI:** 10.3389/fimmu.2013.00034

**Published:** 2013-02-14

**Authors:** Giorgio Santoni, Valerio Farfariello, Sonia Liberati, Maria B. Morelli, Massimo Nabissi, Matteo Santoni, Consuelo Amantini

**Affiliations:** ^1^Section of Experimental Medicine, School of Pharmacy, University of CamerinoCamerino, Italy; ^2^Department of Urology and Andrology, University of PerugiaPerugia, Italy; ^3^Department of Molecular Medicine, Sapienza University of RomeRome, Italy; ^4^Department of Medical Oncology, Polytechnic University of the Marche RegionAncona, Italy

**Keywords:** transient receptor potential, transient receptor potential vanilloid type-2, macrophages, mastocytes, T cell activation, B cell activation, immuno-mediated-diseases, immunomodulation

## Abstract

The transient receptor potential vanilloid type-2 (TRPV2), belonging to the transient receptor potential channel family, is a specialized ion channel expressed in human and other mammalian immune cells. This channel has been found to be expressed in CD34^+^ hematopoietic stem cells, where its cytosolic Ca^2^^+^ activity is crucial for stem/progenitor cell cycle progression, growth, and differentiation. In innate immune cells, TRPV2 is expressed in granulocytes, macrophages, and monocytes where it stimulates fMet-Leu-Phe migration, zymosan-, immunoglobulin G-, and complement-mediated phagocytosis, and lipopolysaccharide-induced tumor necrosis factor-alpha and interleukin-6 production. In mast cells, activation of TRPV2 allows intracellular Ca^2^^+^ ions flux, thus stimulating protein kinase A-dependent degranulation. In addition, TRPV2 is highly expressed in CD56^+^ natural killer cells. TRPV2 orchestrates Ca^2^^+^ signal in T cell activation, proliferation, and effector functions. Moreover, messenger RNA for TRPV2 are expressed in CD4^+^ and CD8^+^ T lymphocytes. Finally, TRPV2 is expressed in CD19^+^ B lymphocytes where it regulates Ca^2^^+^ release during B cell development and activation. Overall, the specific expression of TRPV2 in immune cells suggests a role in immune-mediated diseases and offers new potential targets for immunomodulation.

## TRPV2: A MEMBER OF THE TRP CHANNEL FAMILY

**FIGURE 1:**
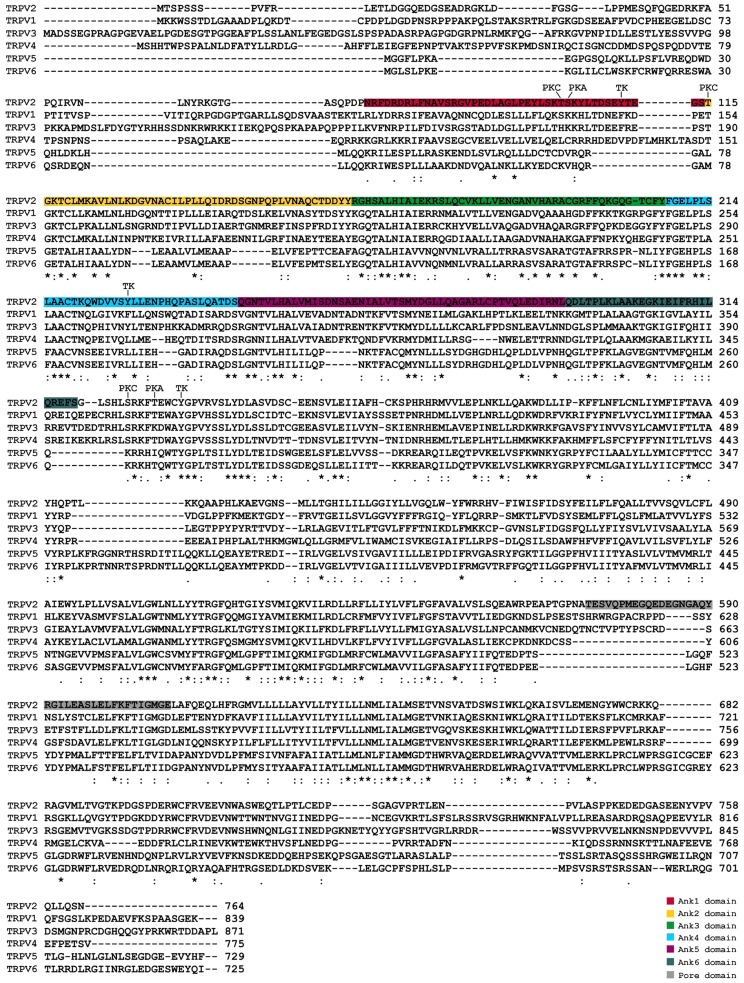
**Alignment and comparison of human TRPVs amino acid structure, highlighting the known functional domains and the potential phosphorylation sites**. PKA, protein kinase A; PKC, protein kinase C; TK, tyrosine kinase.

The 30 mammalian transient receptor potential (TRP) cation channels identified so far can be sorted into seven subfamilies: TRPC (canonical), TRPM (melastatin), TRPV (vanilloid), TRPA (ankyrin transmembrane protein), TRPP (polycystin), TRPML (mucolipin), and TRPN (NomPC-like). TRPs are essentially classified according to their primary amino acid sequence rather than selectivity or ligand affinity. From a structural standpoint, TRP channels are membrane proteins with six putative transmembrane spans (TMs) and a cation-permeable pore region formed by a short hydrophobic stretch between TM5 and TM6 (Owsianik et al., 2006; **Figure 1**). TRP proteins are essentially cation-permeable ion channels sensitive to a remarkable range of stimuli. Genetic approaches in worms, flies, and mice have demonstrated the involvement of TRPs in a variety of sensory processes that include thermosensation, osmosensation, olfaction, taste, mechanosensation, vision, and pain perception. Remarkably, mutations in different TRPs have also been linked to human diseases (Nilius et al., 2007).

Among TRPV channels, TRPV2 is a non-selective cation channel showing Ca^2+^ permeability (Caterina et al., 1999). Its activation triggers an inward cation current (mainly Ca^2+^) that mediates a relatively broad repertoire of physiological functions in response to noxious heat, with an activation threshold of >52°C, as well as to changes in osmolarity and membrane stretch. Human TRPV2 (hTRPV2) channel is triggered by agonists such as ∆^9^-tetrahydrocannabinol (∆^9^-THC) and cannabidiol (CBD; Neeper et al., 2007; Qin et al., 2008; Nabissi et al., 2013). It was found to be expressed both in the plasma membrane and early endosome. Activation of TRPV2 by growth factors causes PI-3K-dependent and independent translocation in the plasma membrane (Kanzaki et al., 1999; Penna et al., 2006). In addition, TRPV2 may serve as an endosomal calcium release channel that controls endosome fusion and/or exocytosis (Saito et al., 2007; Abe and Puertollano, 2011). It has been demonstrated that TRPV2 expression and activity are increased following inflammation, by the action of growth factors such as insulin-like growth factor I (IGF-I; Shimosato et al., 2005). However, the contribute of TRPV2 to inflammation requires further investigations.

This work summarizes data reported in the literature on the expression and function of TRPV2 in hematopoietic stem cells (HSCs) and in natural and adaptive immune cells (**Table 1**).

**Table 1:** Role of TRPV2 in immune cells.

Cell type	Species	TRPV2-mediated effect	Reference
Neutrophils	Human	Migration	Heiner et al. (2003)
Monocytes/ macrophages	Mouse	Migration	Nagasawa et al. (2007)
	Mouse	Phagocytosis	Link et al. (2010)
	Mouse	Cytokine production?	Yamashiro et al. (2010)
	Mouse	Differentiation	Kajiya et al. (2010)
Mast cells	Human	Degranulation	Zhang et al. (2012)
T cells	Human	T cell receptor and Ca^2^^+^ signaling	Sauer and Jegla (2006)

## EXPRESSION OF TRPV2 IN HSCs

Stem cells are found in all multi-cellular organisms and are characterized by the ability to self-renew through mitotic cell division and differentiate into a range of specialized cell types. Multiple functional ion channel currents have been reported to be heterogeneously present in different types of stem cells. They include the voltage-gated delayed rectifier K^+^ current (IKDR), the Ca^2+^-activated K^+^ current (KCa), inward rectifier K^+^ current, hyperpolarization-activated cyclic nucleotide regulated cation current, chloride current, voltage-gated Na^+^ current, L-type calcium current, and TRP non-selective cation currents (Li and Deng, 2011).

While these channels are key players in the pathophysiology of excitable cells, a wide variety of ion channels are also expressed by non-excitable cells, such as cells of the immune system, where they function in signaling pathways regulating electrolyte transport, cell volume, proliferation, differentiation, and apoptosis.

CD34^+^ HSCs give rise to all types of blood cells from the myeloid (monocytes, neutrophils, erythrocytes, dendritic cells, etc.) and lymphoid lineages [T cells, B cells, and natural killer (NK) cells].

**FIGURE 2:**
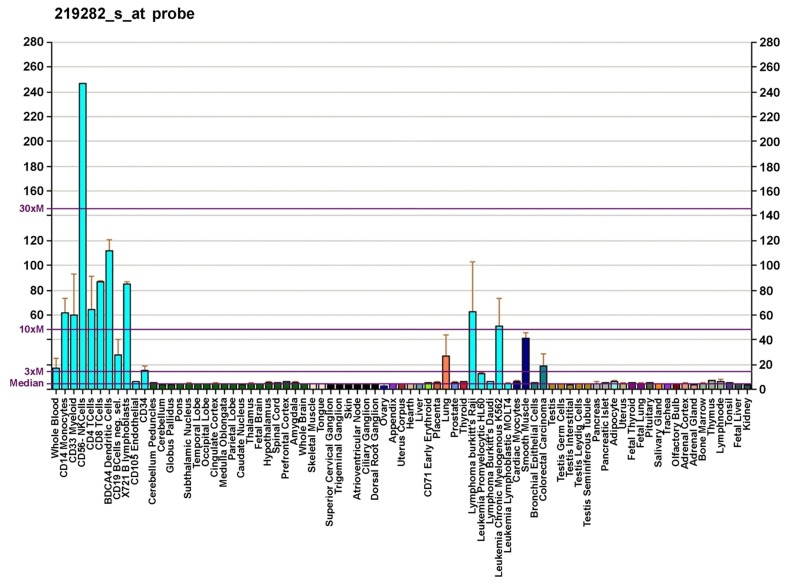
**Gene expression pattern of the TRPV2 gene**. Diagram created by AndrewGNF based on data from Su et al. (2004).

Cytosolic Ca^2+^ activity is crucial for stem/progenitor cell cycle progression and growth (Ferreira-Martins et al., 2009; Resende et al., 2010). Primary human CD34^+^ HSCs express voltage-gated K^+^ channels, two-pore domain background K^+^ channels, and TRP non-selective cation channel family. By using fluorescent-activated cell sorting (FACS) and reverse transcriptase-polymerase chain reaction (RT-PCR), it has been found that human CD34^+^/CD45^+^/CD133^+^CD73^-^ HSCs express TRPV2 channels (Park et al., 2011). Despite the limitation in applying higher temperature conditions, a 42°C TRPV2-like current was consistently observed in CD34^+^ HSCs. These data are in accordance with the previously reported transcriptoma analysis evaluated by *GNF* gene expression (Su et al., 2004) suggesting a peculiar expression of *TRPV2* mRNA in CD34^+^ HSCs (**Figure 2**). Recently, we have demonstrated, both at mRNA and protein levels, that human neural stem cells (NSCs) and glioblastoma stem-like cells (GSCs) express TRPV2 (Morelli et al., 2012), as reminiscence of the primitive myeloid progenitors (Ginhoux et al., 2010).

## TRPV2 CHANNELS IN CELLS OF NATURAL IMMUNITY

Innate immunity, also called natural or native immunity, consists of cellular and biochemical defense mechanisms in place before infection and poised to respond rapidly to infections. The natural immune system includes cells of both myeloid and lymphoid origin, monocytes, macrophages, granulocytes, dendritic cells, and mastocytes as well as NK cells. These cells express many channels belonging to the TRP channel family. Among these, we focused our attention on the expression and function of TRPV2.

### GRANULOCYTES

Immune cells kill microbes by engulfing them in a membrane-enclosed compartment, the phagosome. Phagocytosis is initiated when foreign particles bind to receptors on the membrane of phagocytes. The best-studied phagocytic receptors, those for immunoglobulins (FcγR) and for complement proteins (CR), activate phospholipase C (PLC) and D (PLD), resulting in the intracellular production of Ca^2+^. The molecules that mediate Ca^2+^ ion flux across the phagosomal membrane are still unknown but likely include the ubiquitous store-operated Ca^2+^ entry (SOCE) channels, ligand-gated chloride channel (LGCC), voltage-gated Ca^2+^ channel (VGCC), and TRP channels.

*TRPV2* mRNA has been demonstrated in human neutrophil granulocytes by RT-PCR (Heiner et al., 2003). In these cells, TRPV2 seems to be of particular importance for the response to chemoattractants, suggesting a role in leukocyte migration.

### MONOCYTES AND MACROPHAGES

*TRPV2* gene expression data identify mRNA expression in human CD33^+^ myeloid cells and CD14^+^ monocytes. RT-PCR and immunoblot analyses have shown that *TRPV2* is the sole member of the TRPV family expressed in mouse macrophages (Yamashiro et al., 2010), both in whole blood and in inflammatory tissues such as mouse peritoneal macrophages (Kim et al., 2003) and mouse osteoclasts (Kajiya et al., 2010). In addition, TRPV2 is expressed in human alveolar macrophages (Kowase et al., 2002).

It has been recently demonstrated (Nagasawa et al., 2007) that the chemotactic peptide fMet-Leu-Phe (fMLP) is able to promote the migration of mouse TtT/M87 macrophages by inducing the translocation of TRPV2 channels. This effect was blocked by an inhibitor of PI3-kinase, LY294002, and pertussis toxin. Moreover, treatment with serum-induced translocation of TRPV2 to the plasma membrane is blocked by transfection of *short-form TRPV2* (s-TRPV2) lacking a pore-forming region and the sixth transmembrane domain. In experiments using whole-cell patch clamp, the Ca^2+^ current in TtT/M87 cells was blocked by the TRP channels inhibitor ruthenium red and transfection of either *s-TRPV2* or *siRNA* for *TRPV2*. fMLP induced a rapid and sustained elevation of cytoplasmic Ca^2+^, that was abolished by removal of extracellular calcium, ruthenium red, and transfection of *s-TRPV2* or *siRNA-TRPV2*. Finally, fMLP-induced migration of macrophages was blocked by ruthenium red or transfection of *s-TRPV2*.

**FIGURE 3:**
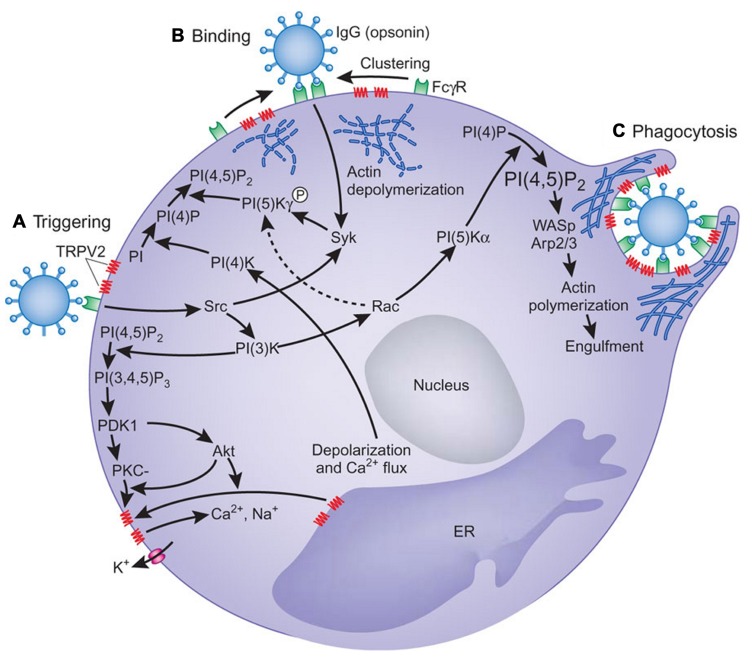
**A model for the involvement of TRPV2 in phagocytosis**. **(A)** Engagement of oligomeric ligands with receptors in the absence of obvious receptor clustering leads to activation of TRPV2 in a PI(3)K-dependent manner, which results in membrane depolarization. How signals downstream of PI(3)K activate TRPV2 remains unclear. Phosphorylation of TRPV2, oligomerization of TRPV2, or recruitment of TRPV2 from intracellular compartment may trigger TRPV2 activation. **(B)** PtdIns(4,5)P2 (PI(4,5)P2) is synthesized by a combination of type II PI(4)K activated by membrane polarization and PIP(5)Kγ activated by Syk. PtdIns(4,5)P2 recruits actin-binding proteins for local actin depolymerization to change local plasma membrane fluidity, thus allowing FcγRs to move. **(C)** Higher order FcγR clustering strengthens the FcγR-mediated signaling, resulting in the activation of PIP(5)Kα and the generation of more PtdIns(4,5)P2, which is sufficient for actin polymerization with the aid of Wiskott–Aldrich syndrome protein (WASp) and Arp2/3. Actin polymerization induces pseudopodia and phagosomal cup formation. PtdIns(4,5)P2, FcγR, and TRPV2 are concentrated in the phagosomal cup. PI, phosphoinositide; PI(4)P, phosphatidylinositol-4-phosphate; PI(3,4,5)P3, phosphatidylinositol-3,4,5-trisphosphate; PKC, protein kinase C; ER, endoplasmic reticulum. From Koyasu (2010). Copyright 2010 Nature Publishing Group.

A role of TRPV2 in early phagocytosis and its fundamental importance in innate immunity was demonstrated in mice by Link et al. (2010). They showed that zymosan-, immunoglobulin G (IgG)-, and complement-mediated particle binding and phagocytosis were impaired in macrophages lacking TRPV2 channels. TRPV2 was recruited to the nascent phagosome and depolarized the plasma membrane. This event increased the synthesis of phosphatidylinositol 4,5-bisphosphate, which triggered partial actin depolymerization necessary for occupancy-elicited phagocytic receptor clustering (**Figure 3**). TRPV2-deficient macrophages were also defective in chemoattractant-elicited motility. Finally, TRPV2-deficient mice showed accelerated mortality and greater organ bacterial load when challenged with *Listeria monocytogenes*.

Several studies have indicated that intracellular Ca^2+^ is a second messenger in Toll-like receptor 4 (TLR4)-dependent signaling. Recently, a role of TRPV2 in lipopolysaccharide (LPS)-induced cytokine mRNA production in mouse macrophages has been demonstrated (Yamashiro et al., 2010). Thus, shRNA against TRPV2 inhibited the LPS-induced mRNA for tumor necrosis factor-alpha (TNF-α) and interleukin (IL)-6 and induced inhibitor of nuclear factor-kappaB (NF-κB) alpha (IkBα) degradation. Experiments using 1,2-bis-(o-Aminophenoxy)-ethane-N,N,N′,N′-tetraacetic acid, tetraacetoxymethyl ester (BAPTA/AM) and ethylene glycol tetraacetic acid (EGTA), and Ca^2+^ imaging indicated that LPS-induced increase in [Ca^2+^]i involves both TRPV2-mediated intracellular and extracellular Ca^2+^ mobilizations. In addition to Ca^2+^ mobilization through the IP3-receptor, TRPV2-mediated intracellular Ca^2+^ mobilization is involved in NF-κB-dependent TNF-α and IL-6 expression, while extracellular Ca^2+^ entry is involved in NF-κB-independent IL-6 production. Another study carried out in TRPV2-knockout (KO) mice (Link et al., 2010) indicates that TRPV2 is not required for LPS-evoked TNF-α protein release. The discrepancy between these two studies could reside in the transcriptional (Yamashiro et al., 2010) or post-transcriptional mechanisms involved in the regulation of cytokine induction.

Recently, it has been demonstrated that the receptor activator of NF-κB ligand (RANKL) induces TRPV2 expression and regulates mouse osteoclast differentiation (osteoclastogenesis) via calcium oscillations and activation of the nuclear factor of activated T cells 1 (NFATc1; Kajiya et al., 2010). Ca^2+^ oscillations are a prerequisite for NFAT-dependent transcription. A possible source of Ca^2+^ for calcineurin activation would be Ca^2+^ entry through TRP channels involved the so-called “store-operated Ca^2+^ entry.” By using a DNA microarray, we found that TRPV2 channels are expressed significantly in RANKL-treated RAW264.7 cells (preosteoclasts) compared to untreated cells. RANKL up-regulates TRPV2 expression in preosteoclasts, evokes spontaneous Ca^2+^ oscillations, and a time-dependent transient inward cation current. The TRPV inhibitor ruthenium red and tetracycline-induced *TRPV2* silencing decreased both the frequency of Ca^2+^ oscillations and transient inward currents in RANKL-treated preosteoclasts. Furthermore, suppression of TRPV2 also reduced RANKL-induced NFATc1 expression, its nuclear translocation and osteoclastogenesis.

### DENDRITIC CELLS

At present very few data have been provided on the expression of TRPV2 in dendritic cells in human. *TRPV2* gene expression was identified in human dendritic cells, but no functional data have been provided so far (Su et al., 2004).

### NK CELLS

*TRPV2* gene is 10- to 30-fold more expressed in human CD56^+^ NK cells compared to all the other cells of both innate and adaptive systems (Su et al., 2004). Although this very high expression of *TRPV2* in NK cells is of great interest, the role of these channels is still unknown. In rats, administration of 2.5 mg/kg of a TRPV2 agonist, CBD, increased the total number of NK cells and their percentage (Ignatowska-Jankowska et al., 2009). Finally, on the basis of Link et al. (2010) data obtained in mouse, a role of TRPV2 in natural and antibody-dependent cytotoxicity of NK cells may be hypothesized.

### MAST CELLS

Mast cells are tissue-resident immune effector cells. They respond to diverse stimuli by releasing potent biological mediators into the surrounding tissue, and initiating inflammatory responses that promote wound healing and infection clearance. In addition to stimulation via immunological routes, mast cells also respond to polybasic secretagogues and physical stimuli. Each mechanism for mast cell activation relies on the influx of calcium through specific ion channels in the plasma membrane (Turner et al., 2007).

The expression, surface localization, and oligomerization of TRPV2 protein subunits together with functional coupling of TRPV2 protein to calcium fluxes and proinflammatory degranulation events have been reported in mast cells (Stokes et al., 2004; Freichel et al., 2012). In addition, a novel protein kinase A (PKA)-dependent signaling module containing PKA and a putative A kinase adapter protein, acyl CoA binding domain-containing protein 3 (ACBD3), that interacts with TRPV2, has been demonstrated in human mast cells (Stokes et al., 2004).

A characteristic of TRPV2 is its activation by high noxious temperature; temperatures exceeding 50°C induced a ruthenium red-sensitive current. In addition, laser light of 640 nm or light at 48 mW for 20 min induced current sensitive to SKF96365, a selective inhibitor of receptor-mediated Ca^2+^ entry and voltage-gated Ca^2+^ entry. Thus, all the three physical stimuli able to activate the TRPV2 channel-induced pronounced degranulation in human mast cells, which could be blocked by ruthenium red or SKF96365. Activation of TRPV2 allows Ca^2+^ ions to enter the cell, which in turn induce degranulation, suggesting that TRPV2 plays a key role in mast cell degranulation in response to mechanical, heat, and red laser light stimulation (Zhang et al., 2012).

## TRPV2 CHANNELS IN ADAPTIVE IMMUNITY

### T LYMPHOCYTES

Calcium acts as a second messenger in many cell types, including lymphocytes. Resting lymphocytes maintain a low concentration of Ca^2+^ (Cahalan and Chandy, 2009; Vig and Kinet, 2009). A network of six distinct types of ion channels, namely Kv1.3, KCa3.1, olfactory receptor class A related 1 (Orai1), stromal interacting molecule 1 (STIM1), Ca^2+^ release activating Ca^2+^ (CRAC) channel, TRPV7 and TRPV2, orchestrates T cell activation, proliferation, and effector functions, offering potential targets for immunomodulation (Sauer and Jegla, 2006; Cahalan and Chandy, 2009). Most recently, TRPV2 has been found to cluster at the immunological synapse following contact with antigen-presenting cells, together with Kv1.3, KCa3.1, STIM1, and Orai1 channels (Sauer and Jegla, 2006; Lioudyno et al., 2008; Cahalan and Chandy, 2009). In regard to TRPV2, Sauer and Jegla (2006) have reported that knockdown of TRPV2 in T cells impairs T cell receptor (TCR) and calcium signaling. Specifically, in Jurkat cells nucleofected with shDNA against hTRPV2, a defect in TCR or thapsigargin-induced calcium mobilization, with predominant effect on the sustained phase of calcium influx, has been reported. Similar to the effect induced by the knockdown of *Lck* (lymphocyte-specific protein tyrosine kinase shDNA), an essential mediator of TCR signaling, *hTRPV2* shDNA transfected cells displayed a reduction in store release, indicative of impaired conformational coupling between CRAC channels and IP3 receptors (Cui et al., 2002). In addition, dominant negative hTRPV2 inhibits endogenous channels mediating Ca^2+^ influx (Sauer and Jegla, 2006).

By quantitative RT-PCR (qRT-PCR), TRPV2 mRNA was detected in whole peripheral blood, primary human T cells (Saunders et al., 2007; Schwarz et al., 2007), CD4^+^ and CD8^+^ T cells (Spinsanti et al., 2008). In rats, administration of the specific TRPV2 agonist, CBD, at a dose of 5 mg/kg caused a significant fall in T cells and T helper (Th) and cytotoxic T (Tc) lymphocyte subsets (Ignatowska-Jankowska et al., 2009). Moreover, CBD decreased the constitutive production of IL-8, macrophage inflammatory protein 1 alpha (MIP-1α and β), and Rantes, and phorbol ester stimulated production of TNF-α, granulocyte-macrophage colony-stimulating factor (GM-CSF), and interferon-gamma (IFN-γ) by human NK cells (Srivastava et al., 1998). In addition, exposure of human T cells THC decreased steady-state levels of mRNA encoding for *Th1* cytokines, while increasing mRNA levels for *Th2* cytokines (Yuan et al., 2002).

### B LYMPHOCYTES

At present, the literature offers very little information on the expression of *TRPV2* mRNA and protein in human. Using GNF gene analysis, the expression of *TRPV2* mRNA was found in whole blood, lymph nodes and tonsils, and human CD19^+^ B lymphocytes (Su et al., 2004). These data were recently confirmed by Boyd et al. (2009), who used qRT-PCR, immunofluorescence and FACS analysis to demonstrate both at mRNA and protein levels the expression of TRPV2 in normal human CD19^+^ B lymphocytes and CD138^+^ plasma cells.

Information about the expression and function of TRPV2 in B lymphocytes was provided by Kanzaki et al. (1999) on growth factor-regulated channel (GRC). It has been shown to be 79.4% identical to mouse TRPV2 (mTRPV2) at the amino-acidic sequence. GRC belongs to the TRP channel family (mTRPV2) localizes mainly in intracellular pools under basal conditions. Upon stimulation of cells by IGF-I, GRC translocates to the plasma membrane. Thus, IGF-I augments calcium entry through GRC by regulating trafficking of the channel (Kanzaki et al., 1999).

**FIGURE 4:**
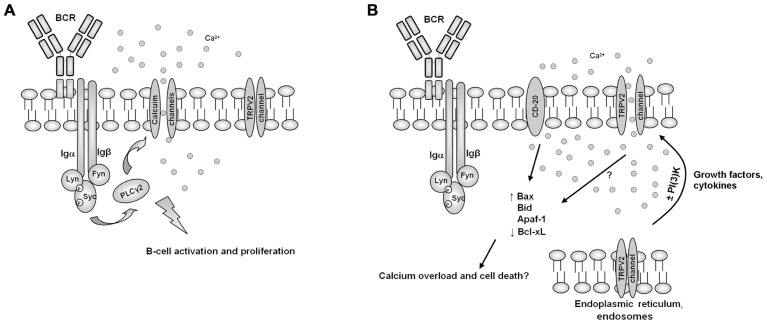
**A model for the involvement of TRPV2 B cell activity regulation**. **(A)** In normal conditions, B cell receptor (BCR) activation induces calcium channels translocation and activation, resulting result in B cell activation and proliferation. **(B)** Growth factors or cytokines, by inducing PI(3)K-dependent or independent pathways, may induce TRPV2 translocation and activation, resulting in calcium overload, B cell activation shut-down and induction of pro-apoptotic cell death pathways, in a similar way to CD20.

Overall, it is conceivable that TRPV2 acts as a transmembrane protein expressed on the surface of B cells, and that it negatively controls Ca^2+^ flux-activated proliferative signal transduction pathways and B cell activation at immunological synaptic level. Thus, the inhibitory role showed by TRPV2 in *in vivo* B cell number, could be the result of CBD-induced translocation of TRPV2 from the cytosol to the plasma membrane (Kanzaki et al., 1999; Ignatowska-Jankowska et al., 2009; Morelli et al., 2012). In the same view, the structural and functional similarity of mTRPV2 with CD20, a calcium permeable cation channel involved in B cell activation (Bubien et al., 1993; Tedder and Engel, 1994; **Figure 4**) and the inhibition of human embryonic kidney 293 (HEK-293) cell proliferation induced by *TRPV2* transfection, further support this hypothesis (Penna et al., 2006; Nabissi et al., 2010).

It has also been suggested that TRPV2 plays an important role in regulating Ca^2+^ release during B cell development. Thus, the promoter of TRPV2 shows binding sites for regulatory transcription factors such as AP2rep, NF-AT1, NF-AT2, and NF-AT3 as well as for Bach-2 (Su et al., 2004), which is critical for class switch recombination and somatic hypermutation of immunoglobulin genes (Muto et al., 2004).

TRPV2 channel has been found to be associated with the recombinase gene activator protein during biosynthesis and early trafficking; it has been observed that over-expression of RGA protein potentiates basal surface localization of TRPV2 and cyclic adenosine monophosphate (cAMP) signal in human non-sensory cells (Barnhill et al., 2004; Stokes et al., 2005). In developing B cells, expression of surface immunoglobulin is an important signal to terminate recombinase activator gene (RAG) expression and V(D)J recombination. Cannabinoids play a critical role in B cell activation and maturation, and the direct role of these compounds in inducing B cell class switching from IgM to IgE has been demonstrated in mice (Agudelo et al., 2008), however, the role of TRPV2 has not been addressed so far.

## CONCLUSION

The TRPV2 is a specialized ion channel expressed in mammalian innate and adaptive immune system. Recent findings on the TRPV2-mediated migration and phagocytosis of granulocytes and macrophages, PKA-dependent mast cell degranulation and its hypothesized role in NK cell cytotoxicity, strongly suggest a major role played by TRPV2 in the control of innate immune responses. Moreover, research on the involvement of TRPV2 as negative transductor in T and B cell activation is still at the beginning. One recent finding is the expression of TRPV2 in a chronic inflammatory skin diseases of unknown etiology called erythematotelangiectatic and papulopustular rosacea (Sulk et al., 2012).

Genetic approaches are required to advance a causal understanding on the role of TRPV2 in inflammatory immune-mediated diseases and cancer. These approaches include over-expression of dominant negative variants, antisense oligonucleotides, and siRNA.

Moreover, although the search for natural TRPV2 ligands and chemical modulators as therapeutic agents has been intensified in the last years (e.g., synthetic and endogenous cannabinoids), specific agonists or blockers and a specific monoclonal anti-hTRPV2 antibody are still lacking.

Further study should be performed to completely address the role of TRPV2 in the pathophysiology of the immune system.

## Conflict of Interest Statement

The authors declare that the research was conducted in the absence of any commercial or financial relationships that could be construed as a potential conflict of interest.
